# Worldwide research productivity on tramadol: a bibliometric analysis

**DOI:** 10.1186/s40064-016-2801-5

**Published:** 2016-07-19

**Authors:** Waleed M. Sweileh, Naser Y. Shraim, Sa’ed H. Zyoud, Samah W. Al-Jabi

**Affiliations:** Department of Pharmacology/Toxicology, College of Medicine and Health Sciences, An-Najah National University, Nablus, 44839 Palestine; Department of Pharmaceutical Chemistry and Technology, College of Medicine and Health Sciences, An-Najah National University, Nablus, 44839 Palestine; Department of Clinical and Community Pharmacy, College of Medicine and Health Sciences, An-Najah National University, Nablus, 44839 Palestine

**Keywords:** Bibliometric, Tramadol, Scopus, Pain management

## Abstract

**Background:**

Pain management and safe use of analgesics is an important medical issue. Tramadol is an old analgesic with controversial properties. Evaluation of worldwide scientific output on tramadol has not been explored. Therefore, the main objective of this study was to give a bibliometric overview of global research productivity on tramadol.

**Methods:**

SciVerse Scopus was used to retrieve and quantitatively and qualitatively analyze worldwide publications on tramadol.

**Results:**

A total of 2059 original and review research articles on tramadol were retrieved from Scopus. Forty-six documents (2.23 %) were published in *Anesthesia and Analgesia* Journal whereas 30 (1.46 %) were published in *Arzneimittel Forschung Drug Research* Journal. Retrieved tramadol documents were published from 71 countries and appeared in 160 peer reviewed journals. Although the United States of America (259; 12.86 %) had the largest contribution to tramadol publications; the contribution by other countries like Turkey (232; 11.27) India (189; 8.09 %) and Germany (176; 8.56 % was not far away from that of USA. The most productive institution was Grunenthal, Germany (47; 2.28 %) followed by Tehran University of Medical Sciences, Iran (29; 1.41 %), and, Ortho-McNeil Pharmaceutical Incorporated, USA (25; 1.21 %). Of the 2059 documents, there were 370 documents about dependence. The leading institution in documents pertaining to tramadol dependence was Grunenthal GmbH (18; 4.86 %) followed by Ortho-McNeil Pharmaceutical Incorporated (17; 4.59 %).

**Conclusions:**

The current study showed that there is an obvious interest in tramadol research. More efforts are needed to clarify the abuse potential and safety profile of tramadol to help in determining the legal status of tramadol. Collaboration among pharmaceutical industry, clinical researchers and academic institutions can improve research quantity and quality on tramadol.

## Background

Search for best methods of pain management is as old as mankind (Gildenberg 1997; Greenblatt et al. 1997). However, only in the past several decades did research about pain management showed great momentum and became a cornerstone in the medical field. The initiation of several international peer reviewed journals specialized in the field of pain/anesthesia has contributed to this positive momentum in pain research. Furthermore, the discovery and development of potent and unique analgesics have also increased attention to pain research. No doubt that the increase in prevalence of chronic pain has also stimulated many pharmaceutical companies to search for new analgesic medications those bear ideal characteristics. Despite the ongoing research and development in the field of pain, no ideal analgesic drug has been found yet. A study that discussed the characteristics of more than 50 analgesics introduced in the past 50 years concluded that despite intense research efforts, there is no real novel analgesic drug discovery and an ideal analgesic is not yet available (Kissin 2010). In 1977, tramadol, a new and atypical opioid analgesic medication, was introduced and marketed by the German pharmaceutical company, Grünenthal GmbH. Since that time, many articles have been published about tramadol and its unique pharmacology and toxicology. Tramadol is unique in that it affects serotonin and norepinephrine systems in addition to its weak μ-opioid receptor agonist activity (Dayer et al. 1994, 1997; Grond and Sablotzki 2004; Epstein et al. 2006; Raffa 2008; Reeves and Burke 2008). Therefore, tramadol has dual mechanism of action; µ opioid agonist and a serotonin/norepinephrine reuptake inhibitor (Raffa 2013; Vazzana et al. 2015). That is why tramadol causes adverse effects pertaining to elevated brain serotonin levels (serotonin syndrome) especially when co-administered with selective serotonin reuptake inhibitors (SSRI) drug class (Nelson and Philbrick 2012; Beakley et al. 2015). Another important pharmacological aspect of tramadol is its metabolism by CYP2D6 which exhibits wide polymorphism leading to variation in toxic and therapeutic effects of tramadol (Lassen et al. 2015; Ibrahim et al. 2016).

The abuse potential of tramadol is still an ongoing controversy (Friderichs et al. 1978; Murano et al. 1978; Richter et al. 1985; Ehrenreich and Poser 1993; Liu et al. 1999; Aknine et al. 2000; Yates et al. 2001; Brinker et al. 2002; Ripamonti et al. 2004; Naslund and Dahlqvist 2003; Woody et al. 2003; Skipper et al. 2004; Soyka et al. 2004). Understanding progress made in pain management research in general and about tramadol in particular requires periodic assessment of how scientific research in this topic is advancing. Bibliometric analysis, a statistical method for literature evaluation, is commonly used for assessment of research activity in any particular filed (Wallin 2005). Several bibliometric studies on pain related topics have been recently published (Robert et al. 2008a, b; Dubner 2009; Mogil et al. 2009; Onyeka and Chukwuneke 2014). However, based on author’s best knowledge, no bibliometric analysis has been published about analgesic drugs, particularly about a controversial medication like tramadol. In this study we planned to give a bibliometric overview of tramadol publications through analysis of temporal research productivity and analysis of highly cited articles in tramadol field. This study is important for those in the field of clinical pharmacology and medicine since it will give them a comprehensive look on current research trends on tramadol and future status of pain management in general and role of tramadol in pain management guidelines.

## Methods

In this study, traditional bibliometric indicators were applied and presented. Such indicators have been explained in previous studies by the same authors. Standard competition ranking (SCR) was used to rank productive journals, countries, authors and institutions. The *h*-index (Hirsch 2005) was used to assess quantity and quality of research productivity by authors, countries and institutions. The journal impact factor (IF) obtained from the Journal Citation Report (JCR; Web of Knowledge) 2014 science edition by Thomson Reuters and the *SCImago Journal Rank* (SJR) indicator (available at: http://www.scimagojr.com/SCImagoJournalRank.pdf) were used as a quality indicator for journal strength and reputation. The adjustment index (AI) was used to stratify productivity with income and population size and was obtained using the following equation: “AI = [total number of publications for the country/gross domestic product (GDP) per capita of the country] × 1000, where the GDP per capita = GDP/population of the country” (Sweileh et al. 2013; Zyoud et al. 2014e, 2015a). In this study, the keyword entered into Scopus search engine was “tramadol” in the “Article Title”. All subject areas were selected and the time interval of the analysis was set up to December 31st, 2013. Analysis was restricted to original and review articles. Citation analysis was completed within 1 day on July 29th, 2015 to avoid bias due to daily in database update. The extracted data were tabulated and analyzed according to the indicators found in previous bibliometric studies (Sweileh et al. 2014; Zyoud et al. 2014b, c, e, f, 2015a, d).

## Results

A total of 2429 research articles on tramadol were retrieved. Of the 2429 articles, there were 1958 (80.61 %) original journal articles, 101 (4.16 %) review articles, 146 (6.01 %) letters, 67 (2.76 %) conference papers, and 157 (6.47 %) other types of publications such as editorial, notes, book chapters, erratum and undefined. Analysis in this manuscript was based on 2059 documents that were original research articles and reviews. The 2059 articles were written in 23 different languages. The main language encountered was English (n = 1687; 80.68 %) followed distantly by Turkish (n = 87; 4.16 %), Chinese (n = 72; 3.44 %), German (n = 62; 2.97 %), Spanish (n = 56; 2.68 %), French (n = 24; 1.15 %), Italian (n = 21; 1.00 %), and 16 other languages (82; 3.92 %). The number of published documents about tramadol in the past 5 years (2008–2013) has increased by approximately 15-folds compared with the time interval from 1978 to 1988 (Table 1). The first article on tramadol was published in 1978. Retrieved tramadol articles were published from 71 countries and appeared in 160 peer reviewed journals. Top ten productive countries on tramadol research are shown in Table 2. Tramadol research and publications were produced by countries from different world regions and even from countries outside Europe and North America. Although the largest number of articles about tramadol was produced by the USA (259; 12.58 %); the contribution by other countries like Turkey (232; 11.27 %), India (189; 8.09 %) and Germany (176; 8.56 %) was not far away from that of the USA (Table 2). It is noteworthy that Turkey surpassed Germany and other European countries in tramadol research.

**Table 1:** Worldwide growth of tramadol research activity

Time interval	Number of published documents N = 2059	%
2009–2013	778	37.79
2004–2008	586	28.46
2003–1999	383	18.6
1994–1998	198	9.62
1989–1993	63	3.06
<1989	51	2.48

**Table 2:** Top 10 countries in tramadol research

SCR^a^	Country	Articles (%)	Total citation	H-index	Average citation	Median (Q1–Q3) of citation	Collaborations with foreign countries	Number (%)^a^ of documents with international authors	Adjustment index
1st	United States	259 (12.58)	9273	53	19 (9–46)	38.44	26	61 (23.55)	4.74
2nd	Turkey	232 (11.27)	2067	24	3 (1–13)	8.91	4	8 (3.45)	22.00
3rd	India	189 (8.09)	1019	16	2 (0–6)	5.39	5	8 (4.23)	115.85
4th	Germany	176 (8.56)	5533	40	17 (6–37)	31.44	17	36 (20.45)	3.69
5th	China	134 (6.51)	835	16	2 (0–7.25)	6.23	4	10 (7.64)	17.64
6th	Iran	101 (4.90)	1094	18	5 (2–11.5)	10.83	4	8 (7.92)	19.08
7th	Italy	99 (4.81)	1609	22	6 (1–11)	7.01	15	24 (24.24)	2.83
8th	Spain	80 (3.89)	1112	22	8.5 (1–24)	13.90	14	29 (36.25)	2.64
9th	United Kingdom	64 (3.11)	2238	26	19.5 (6.25–41.5)	34.97	15	24 (37.50)	1.40
10th	France	50 (2.43)	952	16	9 (2–20.25)	19.04	13	20 (40.00)	1.17

^a^Percentage of documents with international authors from the total number of documents for each country

*SCR* standard competition ranking, *Q1*–*Q3* lower quartile–upper quartile

The total citations of retrieved articles was 36,275, with an average of 17.62 citations per article as recorded in Scopus on July 29th, 2015. The *h*-index of tramadol publications was 74. List of most commonly cited articles on tramadol is shown in Table 3 (Raffa et al. 1992, 1993; Vickers et al. 1992; Lee et al. 1993; Poulsen et al. 1996; Harati et al. 1998; Sindrup et al. 1999; Scott and Perry 2000; Xia et al. 2000; Grond and Sablotzki 2004). Those about the potential mechanism of action and potential role of tramadol in treatment of neuropathic pain were most commonly cited. Two of the top 10 cited articles were published from the same research group, the Raffa et al., group present in the USA. Table 4 shows top 10 productive journals involved in tramadol research. Forty-six articles (2.24 %) were published in *Anesthesia and Analgesia* whereas 30 (1.46 %) were published in *Arzneimittel Forschung: Drug Research*. Of the top 10 journals, six were in the field of anesthesia and pain, three in the field of pharmacology, therapeutics and drug research and one in the field of clinical pharmacology and anesthesia. Of the top 10 journals, two were Turkish journals while the others were based either in USA or Europe. When the journals were screened for IF, three journals of the top productive ones were not listed in ISI JCR and the remaining were listed and had an IF <5.

**Table 3:** Top 10 cited articles in tramadol-related research

SCR^a^	Authors	Journal	Cited by
1st	Raffa et al. (1992)	*Journal of Pharmacology and Experimental Therapeutics*	802
2nd	Harati et al. (1998)	*Neurology*	416
3rd	Grond and Sablotzki (2004)	*Clinical Pharmacokinetics*	402
4th	Lee et al. (1993)	*Drugs*	375
5th	Xia et al. (2000)	*Bulletin of Hunan Medical University*	360
6th	Scott and Perry (2000)	*Drugs*	331
7th	Raffa et al. (1993)	*Journal of Pharmacology and Experimental Therapeutics*	325
8th	Vickers et al. (1992)	*Anaesthesia*	292
9th	Poulsen et al. (1996)	*Clinical Pharmacology and Therapeutics*	290
10th	Sindrup et al. (1999)	*Pain*	273

*SCR* standard competition ranking

^a^Equal articles have the same ranking number and then a gap is left in the ranking numbers

**Table 4:** Top 10 journals in which tramadol-related documents were published

SCR^a^	Journal	N (%) N = 2059	IF	SJR
1st	*Anesthesia and Analgesia*	46 (2.23)	3.472	1.50
2nd	*Arzneimittel Forschung: Drug Research*	30 (1.46)	0.701	0.25
2nd	*Agri:* *The Journal of The Turkish Society of Algology*	30 (1.46)	N/A	0.21
4th	*British Journal of Anaesthesia*	28 (1.36)	4.853	1.84
4th	*European Journal of Pharmacology*	28 (1.36)	2.532	0.87
6th	*European Journal of Anaesthesiology*	27 (1.31)	2.942	0.96
7th	*Paediatric Anaesthesia*	19 (0.92)	1.850	0.84
7th	*Journal of Anaesthesiology Clinical Pharmacology*	19 (0.92)	N/A	0.30
9th	*Clinical Therapeutics*	18 (0.87)	2.731	0.80
9th	*European Journal of Clinical Pharmacology*	18 (0.87)	2.966	1.03
9th	*Egyptian Journal of Anaesthesia*	18 (0.87)	N/A	0.15

*SCR* standard competition ranking, *IF* impact factor, *SJR* standard journal ranking, *N/A* not available

^a^Equal journals have the same ranking number and then a gap is left in the ranking numbers

Table 5 presents scientific subjects of the 2059 articles. As expected, the majority were in the field of medicine (1303; 63.38 %) followed by pharmacology/toxicology/pharmaceutics (721; 35.07 %) and chemistry (189; 9.19 %). Of the 2059 documents, there were 370 documents about dependence. The leading institution in documents pertaining to tramadol dependence was Grunenthal GmbH (18; 4.86 %) followed by Ortho-McNeil Pharmaceutical Incorporated (17; 4.59 %). Table 6 shows top 10 highly productive institutions which published most about tramadol. The most productive institution was *Grunenthal, Germany* (47; 2.44 %) followed by *Tehran University of Medical Sciences, Iran* (25; 1.30 %), and, *Ortho*-*McNeil Pharmaceutical Incorporated, USA* (25; 1.3 %). Table 7 presents the top 10 productive authors on about tramadol, along with their affiliations. Half of the top prolific authors were from USA and seven were affiliated with academic institutions.

**Table 5:** Top 10 subject fields in tramadol-related documents

SCR^a^	Subject area	N (%)^b^ N = 2059
1st	Medicine	1303 (63.38)
2nd	Pharmacology, toxicology and pharmaceutics	721 (35.02)
2nd	Chemistry	189 (9.18)
4th	Biochemistry, genetics and molecular biology	177 (8.60)
5th	Neuroscience	132 (6.41)
6th	Veterinary	119 (5.78)
7th	Environmental science	46 (2.23)
8th	Psychology	42 (2.04)
9th	Agricultural and biological sciences	30 (1.46)
9th	Dentistry	30 (1.46)
9th	Nursing	30 (1.46)

*SCR* standard competition ranking, *USA* United States of America

^a^Equal research areas have the same ranking number and then a gap is left in the ranking numbers

^b^The total % exceeds 100 % because of overlap among subjects

**Table 6:** Top 10 productive institution in tramadol-related research

SCR^a^	Institute	N (%) N = 2059 (100 %)	Affiliation of the institute
1st	Grunenthal GmbH	47 (2.28)	Germany
2nd	Tehran University of Medical Sciences	29 (1.41)	Iran
3rd	Ortho-McNeil Pharmaceutical Incorporated	25 (1.21)	USA
4th	Çukurova Üniversitesi	24 (1.17)	Turkey
5th	R. W. Johnson Pharmaceutical Research Institute	17 (0.83)	USA
6th	Universidade de Sao Paulo	16 (0.78)	Brazil
7th	University of Tennessee, Knoxville	13 (0.63)	USA
8th	Universität Bonn	12 (0.58)	Germany
8th	Università degli Studi di Roma La Sapienza	12 (0.58)	Italy
8th	Poznan University of Medical Sciences	12 (0.58)	Poland
8th	Bethune International Peace Hospital of the PLA	12 (0.58)	China

*SCR* standard competition ranking, *USA* United States of America

^a^Equal institutions have the same ranking number and then a gap is left in the ranking numbers

**Table 7:** Top 10 authors in tramadol research

SCR^a^	Author name	Total number of articles	Affiliation	Country
1st	R. B. Raffa	18	Temple University, School of Pharmacy, Philadelphia	USA
2nd	M. Giorgi	16	University of Pisa, Department of Veterinary Clinical Sciences, Pisa	Italy
3rd	M. Kamin	15	Ortho Biotech Products, L.P., Bridgewater	USA
3rd	N. R. Rosenthal	15	Ortho-McNeil Pharmaceutical Incorporated, Raritan	USA
5th	S. K. Cox	14	University of Tennessee, Knoxville, Department of Biomedical and Diagnostic Sciences, Knoxville	USA
6th	Y. Gunes	13	Cukurova University, Department of Anesthesiology, Adana	Turkey
6th	D. Jordan	13	Ortho-McNeil Pharmaceutical Incorporated, Raritan	USA
6th	D. Ozcengiz	13	Cukurova University, Department of Anesthesiology, Adana	Turkey
9th	G. Saccomanni	12	Universita di Pisa, Dipartimento di Farmacia, Pisa	Italy
9th	K. Minami	12	Emergency Life-Saving Technique Academy of Tokyo, Hachioji	Japan
9th	G. Isik	12	Çukurova University, Faculty of Medicine, Department of Anesthesiology, Adana	Turkey

*SCR* standard competition ranking, *USA* United States of America

^a^Equal authors have the same ranking number and then a gap is left in the ranking numbers

## Discussion

Evidence-based pain management requires exploration of efficacy and safety of various analgesics as presented in literature. Our study analyzed 2059 research articles retrieved from Scopus, a large databases equipped with search options that facilitate citation and comparative analysis (de Granda-Orive et al. 2011). Scopus covers higher number of journals in different subject areas and allows citation analysis which made it very suitable for bibliometric analysis compared to other databases (Tadmouri and Bissar-Tadmouri 2004; Falagas et al. 2008; Kulkarni et al. 2009; de Granda-Orive et al. 2011).

Tramadol was developed in early 1960s by Grunenthal, a German pharmaceutical company. Tramadol entered the West German market in late 1970s with the trade name Tramal^®^. Approximately 20 years later, tramadol was introduced to the US market. Currently, tramadol is available in 100 countries under different trade names. Although tramadol is unique in having dual mechanism of action, its introduction to the worldwide market was slow and cautious. A possible reason for this slow introduction was the uncertainty regarding safety and abuse potential. However, reports and clinical trials have shown that tramadol has lesser toxic profile and better tolerability than oral traditional nonsteroidal anti-inflammatory drugs (NSAIDs) or commonly used opioids (Preston et al. 1991; Adams et al. 2006; Epstein et al. 2006). A study carried out to compare the abuse potential among NSAIDs, codeine, placebo and tramadol has found that abuse potential for tramadol is significantly lower than that of codeine (Adams et al. 2006). On the other hand, reports about other serious side effects like seizure have been reported (Tobias 1997; Raffa and Stone 2008; Sen et al. 2009; Singh et al. 2009; Talaie et al. 2009; Taghaddosinejad et al. 2011; Yarkan Uysal et al. 2011; Boostani and Derakhshan 2012; Mehrpour 2013).

In our study, bibliometric techniques were applied to give an overview on worldwide published research on tramadol. The first publication about tramadol appeared in 1978 followed by rapid increase, particularly after 1995 which coincide with the year of introduction in the US market. The first article about tramadol as analgesic was published by Rost, A., Schenck, E.G. in Arzneimittel-Forschung/Drug Research in 1978 as a comparative clinical study (Rost and Schenck 1978). The discovery and identification of tramadol and its unique mechanism of action lead to the development of a newer tramadol-like agent named tapentadol which is currently available in the market and promoted to have better safety profile than tramadol (Giorgi et al. 2012; Mercadante et al. 2012, 2013; Cepeda et al. 2013; Gohler et al. 2013; Lee et al. 2013; Schwittay et al. 2013; Singh et al. 2013; Fejos et al. 2014).

An average of 17.62 (median = 6) citations per article was obtained in this study. This finding means that tramadol is an interesting topic for scientific research community. Furthermore, the fact that the top 10 productive countries and top 10 productive institutions in tramadol research included countries and institutes outside the Americas and Europe suggest that tramadol research is of interest to many nations. It was obvious that non-academic institutions, particularly pharmaceutical companies, were involved heavily in tramadol research for human or veterinary use (Wu et al. 2001; Teppema et al. 2003; Giorgi et al. 2009a, b). In addition, research about tramadol and its potential use in premature ejaculation gave further momentum to tramadol research (Safarinejad and Hosseini 2006; Salem et al. 2008; Bar-Or et al. 2012). At the official level, research interest in tramadol was motivated by the debate about the legal status and whether to schedule or un-schedule tramadol. In Sweden and in several states in the USA, including New York, Arkansas, Illinois, Kentucky, Mississippi, North Dakota, Oklahoma, Tennessee, Wyoming, and West Virginia have categorized tramadol as a schedule IV controlled substance. In most countries of the world, the legal status of tramadol is currently under re-evaluation.

Finally, the authors believe that this study, like all other bibliometric studies, has few limitations that have been explained in previous bibliometric studies (Li et al. 2011; Sweileh et al. 2013; Zyoud et al. 2014a, d, 2015b, c; Zyoud 2015). Such limitations include the use of Scopus database; the use of “tramadol” word as a title keyword in the search engine; and the restriction of analysis to original articles and article reviews. However, despite all this, the current study will be of great value for people in pain management policy and those involved in research pertaining to analgesia.

## Conclusions

Tramadol is an analgesic with controversial properties which made it an appealing topic for researchers from different scientific disciplines. Research output about tramadol was not confined to particular country. More research efforts are needed to shed light on the abuse potential and the non-analgesic therapeutic benefits of tramadol. Research activity about tramadol needs to be directed toward minimizing its side effects. Such research activity can be achieved through collaboration among people in pharmaceutical industry, clinical practice and academic institutions.
